# Use of a modified Michaelis-Menten equation to estimate growth from birth to 3 years in healthy full term babies

**DOI:** 10.21203/rs.3.rs-2375831/v1

**Published:** 2023-01-12

**Authors:** William Walters, Catherine Ley, Trevor Hastie, Ruth Ley, Julie Parsonnet

**Affiliations:** Max Planck Institute for Developmental Biology; Stanford University; Stanford University; Max Plank Institute for Developmental Biology; Stanford University, Stanford

**Keywords:** Michaelis-Menten equation, growth, birth cohort, height estimation, weight estimation

## Abstract

Mathematical models that accurately describe growth in human infants are lacking. We used the Michaelis-Menten equation, initially derived to relate substrate concentration to reaction rate, and subsequently modified and applied to nonhuman vertebrate growth, to model growth in humans from birth to 36 months. We compared the model results to actual growth values from two pediatric cohorts.

The modified Michaelis-Menten equation showed excellent fit for both infant weight (median RMSE: boys: 0.22kg [IQR:0.19; 90%<0.43]; girls: 0.20kg [IQR:0.32; 90%<0.39]) and height (median RMSE: boys: 0.93cm [IQR:0.53; 90%<1.0]; girls: 0.91cm [IQR:0.50;90%<1.0]). Using permutations of dropped data, few combinations of time points were critical to optimizing model fit.

This modified Michaelis-Menten equation accurately describes growth in humans aged 0–36 months, allowing imputation of missing weight and height values in individual longitudinal measurement series. The pattern of growth in healthy babies raised in resource-rich environments mirrors the saturation curve of a basic enzymatic reaction.

## Introduction

Height, weight and growth are foundational indicators of child health. Growth charts, created by the World Health Organization (1) and the US Centers for Disease Control and Prevention (2), serve as clinical references to evaluate individual pediatric physical sizes and growth rates against population means. These reference ranges represent cross-sectional information from tens to tens of thousands of children per age group. Longitudinal studies, however, demonstrate the unpredictability of individual patterns, with short growth spurts punctuating periods of minimal growth (i.e., a saltatory pattern) (3, 4). Thus growth for an individual child is statistically unique and cannot be reconstructed from group data (5).

Stanford’s Outcome Research Kids (STORK) is a birth cohort recruited in the San Francisco Bay Area, California, designed to evaluate the impact of infections on growth from birth to age 36 months (6). In this project, some infants were missing required time-specific weight measurements. We sought to identify an empirical longitudinal growth model that would provide the best interpolation of missing weight values given only the available weight values for that individual baby - in essence, a function that not only would smooth noisy existent data to fit a line but that also was simple, to avoid overfitting.

A wide variety of non-saltatory growth models in babies and young children have been proposed and compared (7–11). Many include population means (mixed or multilayer models) to identify deviating individuals and most are complex, for example, including multiphasic curves to account for different growth patterns (12). General growth models, including those seeking to verify the “universality” of determinate growth in mammals, rarely appear to have been applied to infant growth to date (13).

The simple Michaelis-Menten equation was originally used in biochemistry to describe how substrate concentration affects the rate of enzyme catalysis (14). The equation was subsequently slightly modified and applied to a wide range of chemical and biological processes, ranging from antibody development to soil microbial activity to tree growth (15–17). The Michaelis-Menten equation also describes growth in fish, birds and mammals of all sizes with extraordinary accuracy (18). To date, however, the Michaelis-Menten equation has not been used to model human growth.

We used a modified Michaelis-Menten equation to estimate each STORK baby’s individual weight curve and evaluate its fit. We then sought to replicate the use of this equation for both weight and height in a large longitudinal sample of healthy babies from the Stanford Medicine Research Data Repository (STARR) and additionally identified those time point combinations essential for best model fit. Finally, we evaluated the accuracy of the modified Michaelis-Menten equation when using growth measures from the first 12 or 24 months to predict weight and height during the second and/or third year of life.

## Methods

### Babies

Detailed methods for the STORK birth cohort and its nested randomized intervention of personal and household cleaning products have been described previously (6). In brief, a multiethnic cohort of mothers and their 138 babies was followed from the second trimester of pregnancy to the babies’ third birthday. Healthy women aged 18–42 years with a single-fetus pregnancy who wished to participate were enrolled. Households were visited every four months after enrollment until the baby’s third birthday, for a total of nine baby visits. At each visit, the weight of the baby in pounds was recorded using a single scale calibrated daily for all babies; if the child was unable to stand on the scale, the mother was weighed with and without the baby in her arms. Medical chart review was performed as available to identify birth weight and birth length and also any weight and height values recorded at well-baby care visits or medical visits for illness. All data were managed in REDCap (19) hosted at Stanford University.

STARR (starr.stanford.edu) contains electronic medical record information including clinical encounters, diagnoses, laboratory test results and pharmacy orders from all pediatric and adult patients seen at Stanford Health Care (Stanford, CA). For this analysis, no specific sample size was requested so STARR staff identified all babies during the period 03/2013–01/2022 who were followed from birth to at least 36 months of age with at least five well-baby care visits over the first year of life. Weight, height and age in days were abstracted for each visit through age three years, along with sex and race/ethnicity if specified. These data were de-identified and provided to study investigators.

### Statistical analysis

All observed weight and height values were evaluated in kilograms (kg) and centimeters (cm), respectively. Any values assessed beyond 1,125 days (roughly 36 months of age) and those clearly identifiable by two independent reviewers as errors (e.g. a substantial loss in height or biologically impossible gain or loss of weight) were excluded. Additionally, any weights assessed between birth and 19 days were excluded, as weight loss often occurs immediately after birth, and approximately 95% of babies return to their birth weight by 19 days (20). Finally, at least five observations across the 36 month period were required: babies with fewer than five weight or height values after the previous criteria were excluded from our analyses.

### Model

We developed our weight model using values from STORK babies and then validated it with values from the STARR babies. Height models were evaluated in the STARR babies only.

The Michaelis-Menten equation is described as follows:

$$\text{v}={\text{V}}_{\max}\left([\text{S}]/\left({\text{K}}_{\text{m}}+[\text{S}]\right)\right)$$

where v is the rate of product formation, V_max_ is the maximum rate of the system, [S] is the substrate concentration, and K_m_ is a constant based upon the enzyme’s affinity for the particular substrate.

For this study the equation became:

P=a1(Age/(b1+Age))+c1

where *P* was the predicted value of weight (kg) or height (cm), Age was the age of the infant in days, and c1 was an additional constant over the original Michaelis-Menten equation that accounted for the infant’s non-zero weight or length at birth. Each of the parameters a1, b1 and c1 was unique to each child and was calculated using a nonlinear least squares (nls) regression model. In our case, weight data were fitted to a model using the statistical language R (version 3.4.0) (21), by calling the formula nls() with the following parameters:

fitted_model<-nIs(weights~(c1+(a1*ages)/(b1+ages)),start=list(a1=5,b1=20,c1=2.5))

where weights and ages were vectors of each subject’s weight in kg and age in days. The default Gauss-Newton algorithm was used. The optimization objective is not convex in the parameters, and can suffer from local optima and boundary conditions. In such cases good starting values are essential: here, the starting parameter values (a1 = 5, b1 = 20, c1 = 2.5) were adjusted manually using the STORK dataset so as to minimize model failures; these tended to occur when the parameter values, particularly for a1 and b1, increased without bound during the iterative steps required to optimize the model. These same parameter values were used for the larger STARR dataset.

The starting height parameter values for height modeling were higher than those for weight modeling, due to the different units involved (cm vs. kg)(a1 = 60, b1 = 530, c1 = 50). Correlations between the c1 parameter and birth weight or birth length for all babies by sex and by study were evaluated using Spearman’s rank coefficient.

Because this was a non-linear model, goodness of fit was assessed primarily via root mean squared error (RMSE) for both weight and height. (Both RMSE and R^2^ are used as measures of model fit; R^2^ has been shown, however, to give artificially high values (close to 1.0) on non-linear data (22). Lower RMSE values indicate better model fit.) The values of RMSE are in the same units as those measured (in this case, kg or cm), and these values can be used as estimates of the deviation in values predicted by the model from the observed values. To evaluate the effect of age on the RMSE, we considered the RMSE for each timepoint and visualized all RMSE vs. age.

### Imputation tests

To test for the importance of different time points to the accuracy of the modified Michaelis-Menten equation models, we limited our analysis to STARR babies with all recommended well-baby visits (12 over three years (23)). Apart from Day 1 when the birth weight and length were assessed, each scheduled visit occurred in a window of days around the expected well-baby visit age (Visit1: Day 1, Visit2: days 20–44, Visit3: 46–90, Visit4: 95–148, Visit5: 158–225, Visit6: 250–298, Visit7: 310–399, Visit8: 410–490, Visit9: 500–600, Visit10: 640–800, Visit11: 842–982, Visit12: 1024–1125). We considered two different sets of these data to evaluate key time points: infants with all scheduled visits in the first year of life (seven total visits) and those with all scheduled visits over the full three year timeframe (12 total visits). We fit these two sets to the model, to identify the baseline RMSE for these subjects. Then, every visit, combination of pairs of visits, and combination of three visits were dropped, so that the impact on RMSE or model failures for particular visits or combination of visits could be evaluated by comparison to the baseline.

### Prediction

We sought to predict age at 36 months (year 3 [Y3]) from growth measures assessed only up to 12 months (Y1) or to 24 months (Y1 + Y2), utilizing the “last value” approach (7). In brief, the last observation for each child (in our case, growth measures at approximately age 36 months), is used to assess the overall model fit, by focusing on how accurately the model can interpolate the measure at this time point. For this analysis, we identified all STARR infants with at least five time points in Y1 and at least two time points in both Y2 and Y3; the selection of these time points was based on maximizing the number of later time points within the constraints of the well-baby visit schedule for Y2 and Y3. The per subject full set of time points (Y1-Y3) was fitted using the modified Michaelis-Menten equation as above and the mean squared error was calculated, acting as the “baseline” error. The model was then run on the subset of Y1 only and of Y1 + Y2 only. To test predictive accuracy of these subsets, the RMSE was calculated using the actual weights or heights versus the predicted weights or heights of the three time series.

All analyses were performed in R 3.4.0 (21) (data available in Supplemental Data Tables). The STORK study was approved by the Stanford IRB (protocol 17756), which also determined that our analysis using STARR data was exempt.

## Results

A total of 126 STORK and 14,817 STARR babies were initially considered in this analysis (Supplemental Fig. 1). After excluding values per protocol, 97 (77.0%) STORK and 14,695 (99.2%) STARR babies had sufficient measurements to be included in the weight analyses. For height, examined only in STARR, 11,655 (78.7%) babies had sufficient measurements to be included in the height analyses.

The sex of infants was similar in both STORK and STARR. STORK babies were slightly heavier than STARR babies both at birth (p = 0.002) and at approximately 36 months (p = 0.05) (Table 1). For the 97 STORK babies, weight values were spread fairly consistently across the 36 months due to the study design; no height measures were obtained. For the 14,695 STARR babies, the number of weight and height timepoints per subject was quite variable (range: weight: 5–15; height: 5–13).

### Weight models

The Michaelis-Menten model was successfully fitted to 94 STORK babies (95.9%) and 14,596 STARR babies (99.3%). Distributions of the model parameters a1 and b1 were right-skewed; the c1 parameter followed a normal distribution and approximated birthweight (Spearman Rho correlation: 0.79, 0.84 and 0.87 for STORK boys, STORK girls and both STARR boys and girls, respectively; difference between median c1 values and median birth weight: 0.27, 0.03, 0.05 and 0.04 kg in STORK boys, STORK girls, STARR boys and STARR girls, respectively) (Supplemental Fig. 2, Supplemental Table 1).

**Figure 1:**
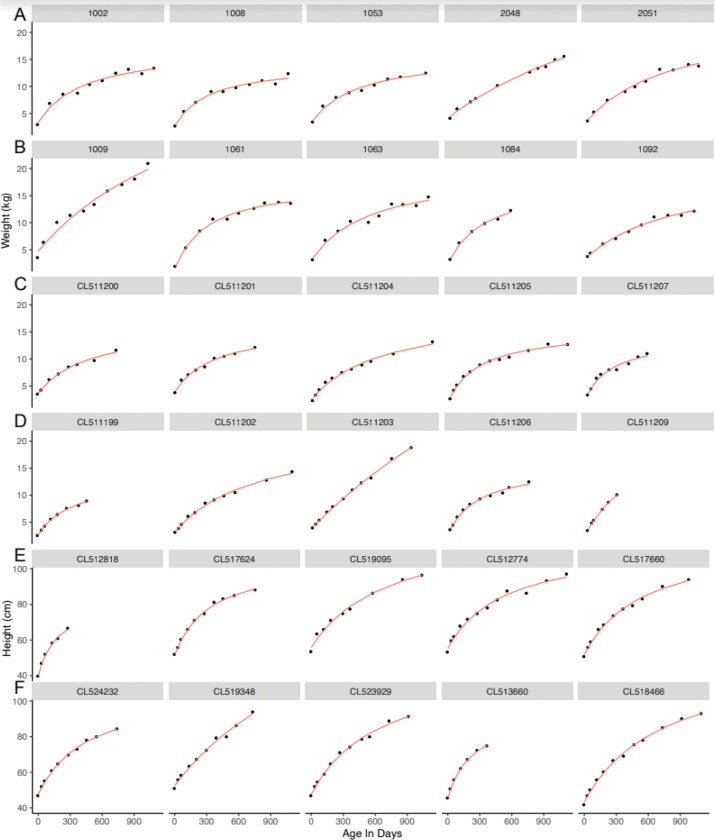
A-F: A representative sample of fitted models for weight (kg) and for height (cm). Weight fitting (in kg) shown for: (A) STORK boys, (B) STORK girls, (C), STARR boys, (D) STARR girls, and height fitting (in cm) for: (E) STARR boys, (F) STARR girls. Each row shows the first five individuals from each given category in the dataset. The red line indicates the fitted model and the black circles indicate actual weights or heights.

**Figure 2:**
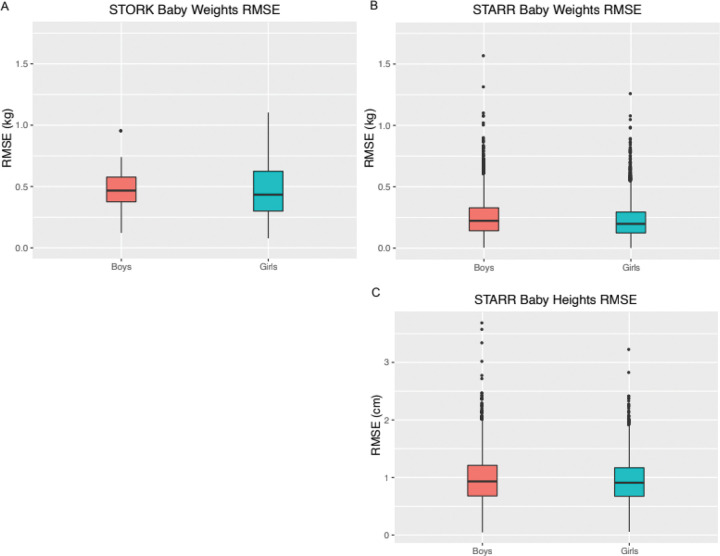
A-C : Distribution of RMSE values for the modified Michaelis-Menten equation in babies by sex for weight (kg) and for height (cm). (A) STORK weights, (B) STARR weights, and (C) STARR heights.

Visual inspection of plots of infant weights over time, along with the fitted curves indicated a good fit with this Michaelis-Menten model for both STORK and STARR babies (Fig. 1, A–D). Accuracies of model fit were high, as measured by low RMSE, particularly in STARR babies (median RMSE, for boys and girls: in STORK: 0.47 and 0.43 kg; in STARR: 0.22 and 0.20 kg; 90% RMSE, for boys and girls, in STORK: 0.65 and 0.74 kg, in STARR: 0.43 and 0.39 kg) (Fig. 2A and B, Supplemental Table 1). Overall, a total of only 11 (0.08%) babies had RMSE values above 1.0 kg (Supplemental Fig. 3); whether these outliers reflect errors in weight value data entry or measurement, or weight loss and gain that deviates from a more typical growth curve, is unknown. The different ethnic and racial groups had similar RMSE values (Table 1, Supplemental Fig. 4). The effect of age on RMSE over time showed a slight increase across three years (Supplemental Fig. 5).

The model failed to fit 4.1% of STORK babies and 0.7% of STARR babies, generally because a1 and b1 parameters increased without bound; these babies tended to show linear, rather than non-linear growth (Supplemental Fig. 6).

### Height models

The model parameters a1 values were slightly left-skewed while the b1 values were right-skewed, with both showing a small number of large outliers; the c1 parameter again had a normal distribution and was correlated with birth length (Spearman Rho: 0.92 and 0.91 for boys and girls, respectively; difference between mean c1 value and birth length: 0.3 cm and 0.5 cm for boys and girls, respectively) (Supplemental Table 1, Supplemental Fig. 7).

Visual inspection of the fitted data for height indicated excellent fit (Fig. 1, E–F) and RMSE values were low for these models, with both median and 90% values under 1 cm (median: 0.93 and 0.91 for boys and girls, respectively; <90%: 0.998 for boys and girls). A total of only five subjects (0.043%) had RMSE over 3 cm (Supplemental Fig. 8). Again, RMSE values were similar across racial and ethnic groups (Supplemental Fig. 4). The effect of age on RMSE over time again showed a slight increase across three years (Supplemental Fig. 5).

Very few subjects (0.25%) failed to fit the model as a1 and b1 parameters increased without bound; these babies showed either very linear growth, or had very large height values that were likely data entry errors rather than aberrant growth (e.g. substantial loss of height) (Supplemental Fig. 9).

### Imputation Tests

In testing critical time points for weight or height imputation, the removal of visit 1 (birth weight or length) increased RMSE more than the removal of any other visit (Supplemental Table 2). When imputing values in the first year alone (visits 1–7), visit 7 at approximately 1 year of age had the second largest impact on model fit. Considering years 1–3, the removal of the final data point alone, visit 12, had a much more modest impact on RMSE, as did the removal of other visits besides visit 1. Most combinations of visits could be dropped, with exceptions: removal of visit 1 in combination with other visits, particularly visits during year 1, led to a sizable increase in RMSE, as did removal of consecutive visits at the final time points (visits 5, 6, and 7 for the year 1 subset; visits 10, 11, and 12 for the years 1–3 subset). The RMSE could be rescued to some degree for missing visit 1 birth data, but not missing final time point data (e.g. visit 7 for year 1 data), by increasing the initial a1 and b1 parameters to higher values (e.g. a1 = 15, b1 = 500).

### Prediction

For weight prediction modeling, a total of 4,829 STARR infants (48.8% female) had at least five time points in Y1 and two in each of Y2 and Y3; of these, 1.8% were dropped due to the inability of the model to fit their growth values (Supplemental Fig. 1). RMSE values were low for the full model (median RMSE: 0.33 and 0.30 kg for STARR boys and girls; Supplemental Table 3). The median RMSE increased to 1.13 and 1.08 kg (boys and girls) when the model predicted Y3 data points using Y1 + Y2 data (Supplemental Fig. 10). Median RMSE increased further to 1.37 and 1.34 kg (boys and girls respectively) when only Y1 data was used to predict fitted data for Y2-Y3.

As with weight data, we tested the Michaelis-Menten equation to predict future heights using models created from Y1 or Y1 + Y2 data (Supplemental Fig. 11). A total of 3,963 STARR infants (49.3% female) had sufficient time points. A small number (0.58%) failed to fit a growth model. Median RMSE values were slightly over 1 cm for complete data fitting (1.14 cm for boys, 1.08 cm for girls) (Supplemental Table 4). Prediction of Y3 data from Y1 + Y2 yielded a RMSE of 2.91 and 2.68 cm for boys and girls, respectively. Median RMSE values for Y2 + Y3 data predicted from Y1 alone were 5.38 cm and 5.63 cm for boys and girls, respectively.

## Discussion

Using longitudinal weight data first in a birth cohort and subsequently a large convenience sample, we found that a modified Michaelis-Menten equation described individual babies’ non-linear growth in weight and height from birth to age 36 months with minimal error. Although certain time points were essential for model accuracy (birth weight or length, and the measure at approximately 12 months), the loss of most other data points had only modest effects on RMSE, indicating that our model can correctly impute weights and heights for infants, even when information from multiple well-baby visits are missing. This equation provides an excellent method to estimate weight or height at any time point within the first three years of life.

The modified Michaelis-Menten equation has been shown previously to describe growth in a wide array of living organisms and in particular mammals, including primates (18). We believe our study is the first to demonstrate its applicability in humans. This equation has the distinct advantage of being conceptually simple: although childhood height and weight are clearly influenced by a multitude of factors, normal growth over time with sufficient resources mirrors an elementary chemical reaction on consumable substrates. Whether this equation is valid for growth in premature babies, babies with severe illness or health conditions or babies in resource-poor environments remains to be determined.

We examined how well the modified Michaelis-Menten equation predicted growth at 36 months and found that estimates based on data from ages 0–24 months were within approximately 2.8% of actual height and 7.5% of actual weight. This difference in precision between height and weight may be because height measurements are less subject to intrinsic variation than weight measurements (24); additionally, but less likely, height might be less prone to measurement error than weight, as children may be weighed with or without clothes. Using measures from only the first year of life to predict height and weight at 36 months was more imprecise (within 5.7% and 9.2% of actual height and weight, respectively). To date, we have found no models designed specifically to predict growth at three years of life; this equation may provide an interesting approach for identifying unexpectedly low or high growth within an individual child up to this age, without focusing on standardized growth curves. Of course, our model includes only the initial sigmoidal growth before age three years; different models should be used when considering other time frames when the growth rate changes significantly (i.e., at puberty).

Limitations of the Michaelis-Menten equation include its inability to handle infants who appear to have linear (vs. non-linear) growth; the proportion of such babies in our study, however, was extremely small (~ 0.7% overall). In this study, we limited our time frame from birth to 36 months; an evaluation of how far along the age spectrum this equation remains reliable would be of interest. It is important to note that body mass index (BMI), a function of height and weight, does not follow a similar curve; this measure however is not typically applicable under the age of two years in the US (25). Finally, while weight and height have been considered useful measures of growth, growth trajectories - their derivatives - are perhaps of greater importance (10, 26, 27).

In summary, a modified Michaelis-Menten equation can accurately describe weight and height in individual, ethnically-diverse infants from birth to age three years in California. Whether this equation can similarly explain growth in premature babies, sick children in resource-poor environments and those in older age categories has yet to be evaluated. Growth over time in an individual baby, similar to that of many known organisms - from bacteria to trees - mirrors the saturation curve of a basic enzymatic reaction.

## Figures and Tables

**Table 1:** Characteristics of STORK and STARR babies

		STORK		STARR	
		N	Statistic ^a^	N	Statistic
Babies in weight analyses		97		14,695	
Babies in height analyses		NA		11,655	
Female		49	50.5	7162	48.7
Birthweight (kg)		96	3.42 (0.46)	14,695	3.28 (0.50)
Birth length (cm)		NA ^b^		11,655	50.23 (2.58)
Weight at ~ 36 months ^c^ (kg)		35	15.48 (2.76)	3,117	14.72 (1.84)
Height at ~ 36 months (cm)		NA		2,514	95.88 (3.79)
Weight measures overall		796	9 (3) [5–10]	133,732	9 (4) [5–14]
Weight measures for ages (months)	0–12	280	3 (1) [3–5]	86,705	6 (0) [4–8]
	13–24	267	3 (1) [1–4]	31,809	3 (2) [0–4]
	25–36	249	3 (1) [0–5]	15,218	1 (2) [0–4]
Height measures overall		NA		107,586	10 (3) [5–13]
Height measures for ages (months):	0–12	NA		68,927	6 (1) [3–8]
	13–24			26,221	3 (1) [0–4]
	25–36			12,438	1 (2) [0–3]
Ethnicity	Hispanic/Latino			1,026	6.9
	Non-Hispanic			8,418	56.8
	Unspecified			5,373	36.3
Race group	Asian			3,220	21.7
	Black			255	1.7
	Native American			18	<1
	Pacific Islander			42	<1
	White			3,911	26.4
	Other			1,858	12.5
	Unspecified			5,513	37.2

a Percent *or* mean (standard deviation [sd]) *or* median (interquartile range [IQR]) [range].

b NA: not applicable in STORK (neither birth length nor height values were ascertained at household visits).

c +/− 2 months.
